# A predicted functional gene network for the plant pathogen *Phytophthora infestans* as a framework for genomic biology

**DOI:** 10.1186/1471-2164-14-483

**Published:** 2013-07-17

**Authors:** Michael F Seidl, Adrian Schneider, Francine Govers, Berend Snel

**Affiliations:** 1 Theoretical Biology and Bioinformatics, Department of Biology, Utrecht University, Padualaan 8, Utrecht, 3584 CH, The Netherlands; 2 Centre for BioSystems Genomics, P.O. Box 98, Wageningen, 6700 AB, The Netherlands; 3 Laboratory of Phytopathology, Wageningen University, P.O. Box 8025, Wageningen, 6700 EE, The Netherlands

**Keywords:** Oomycetes, *Phytophthora infestans*, Protein-protein interaction, Protein-protein associations, Network, Bayesian integration, Protein complex, Functional module

## Abstract

**Background:**

Associations between proteins are essential to understand cell biology. While this complex interplay between proteins has been studied in model organisms, it has not yet been described for the oomycete late blight pathogen *Phytophthora infestans*.

**Results:**

We present an integrative probabilistic functional gene network that provides associations for 37 percent of the predicted *P. infestans* proteome. Our method unifies available genomic, transcriptomic and comparative genomic data into a single comprehensive network using a Bayesian approach. Enrichment of proteins residing in the same or related subcellular localization validates the biological coherence of our predictions. The network serves as a framework to query existing genomic data using network-based methods, which thus far was not possible in *Phytophthora*. We used the network to study the set of interacting proteins that are encoded by genes co-expressed during sporulation. This identified potential novel roles for proteins in spore formation through their links to proteins known to be involved in this process such as the phosphatase Cdc14.

**Conclusions:**

The functional association network represents a novel genome-wide data source for *P. infestans* that also acts as a framework to interrogate other system-wide data. In both capacities it will improve our understanding of the complex biology of *P. infestans* and related oomycete pathogens*.*

## Background

The late blight pathogen *Phytophthora infestans* is one of the most destructive pathogens of tomato and potato, and a continuous threat to global food production [1]. *P. infestans* belongs to the lineage of oomycetes that unites diverse saprophytic and pathogenic species that share morphological similarities to true fungi [2], yet are closely related to non-pathogenic diatoms and brown algae. Over the last two decades, *P. infestans* has gradually developed into a model organism not only for oomycetes, but also for filamentous plant pathogens. The releases of its genome sequence and that of other closely related oomycetes [1,3] have greatly increased our understanding of their complex biology, pathology and evolution (e.g. [4,5]). So far, however, only individual gene products, mostly in the context of pathogenicity, have been intensively studied [6]. Genome-wide experiments elucidating functional associations among proteins have not yet been performed and as a result, the complex interplay of proteins within a cell and its contribution to fundamental cellular processes is poorly understood.

Even though some proteins operate solitarily, the majority is associated with other proteins. They are embedded in a complex network in which assemblies of proteins synergistically mediate a biological function [7,8]. Proteins can associate directly by physical interaction, e.g. in protein complexes, or indirectly, e.g. in the same pathway or cellular process. Functional association networks represent the compendium of all possible associations in a cell. *In vivo*, however, these associations are dynamic and depend on physiological conditions such as external stimuli or changes during the life cycle.

A considerable number of functional association networks in many species have been described: These networks are not only derived from large-scale experimentally determined physical associations [7,9], but also from integrative approaches combining diverse functional and comparative genomics data. Such integrative networks made a substantial contribution in system-wide understanding of the biology of well-studied model organisms such as *Saccharomyces cerevisiae* (budding yeast) and *Arabidopsis thaliana* (thale cress) [10-12]. Many of these studies used a Bayesian framework to integrate heterogeneous data into a single unified network [10,11]: every data source adds a certain level of evidence to the combined evidence of functional linkage between two proteins. At the same time, this approach accounts for differences in the quality of the individual data sources. The resulting network maximizes the coverage of the proteome while ensuring an acceptable level of confidence [11]. The reliability of these integrative approaches has been benchmarked using experimental data that are available in these model organisms. While very few protein-protein interactions or functional associations have been reported in *P. infestans*[13], a considerable amount of transcriptomic and comparative genomic data for *P. infestans* and other related oomycetes is available [1,3,14,15].

In this study, we present the first functional association network in the oomycete model organism *P. infestans.* Our method integrates diverse functional and comparative genomics data sets into a unified network. The first data set is composed of projected interactions based on interolog mapping. Interolog mapping describes the transfer of protein-protein interactions from one organism to another: proteins in the species of interest are expected to interact if their orthologs in another species have been shown to interact [16]. The second data set adds predicted associations between proteins encoded by co-expressed genes [17,18]. Thirdly, we used conserved co-expression, i.e. orthologs of co-expressed genes in one species are also co-expressed in a related species, to increase the moderate predictive power of gene co-expression towards functional association [19]. As a fourth line of evidence we predicted interacting proteins by conserved phylogenetic co-occurrence of the two encoding genes across a considerable amount of divergent species [20]. This approach assumes that interaction partners should either be gained or lost together, as a single interaction partner cannot perform the full function. We adapted a scoring schema that assesses the merit of each individual data set and subsequently integrates the data using a Bayesian approach yielding a comprehensive functional association network, covering 37% of the predicted proteome of *P. infestans*. Our predicted network enables the in-depth analysis of complex omics data such as microarrays. For example, in the predicted functional association network we identified functional modules of differentially expressed genes during distinct life phases of *P. infestans*, thereby highlighting dynamic features of this network. These functional modules place unknown gene products in a cellular context. The functional association network represents a valuable addition to the growing genomic resources for *P. infestans* serving as an important framework for in-depth analyses of existing and yet to appear omics data. We anticipate that its availability will add significant knowledge to our understanding of the complex biology of this devastating plant pathogen.

## Results and discussion

### Adaptation of a Bayesian scoring schema in *P. infestans*

**Figure 1:**
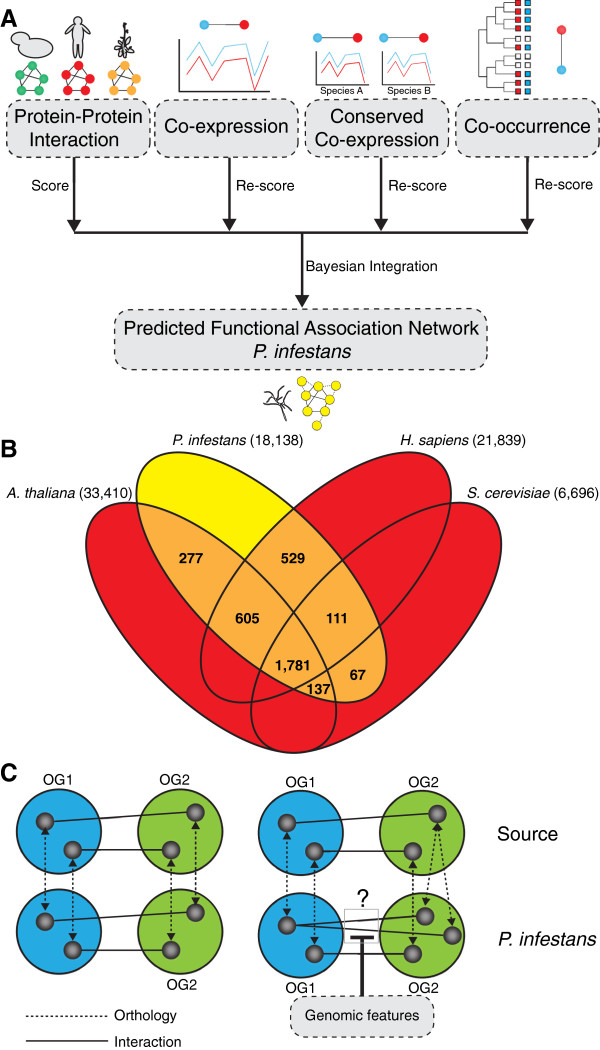
**Prediction of functional association network in *P. infestans. *(A)** Integration of four distinct data sources to predict the functional association network in *P. infestans*. We predicted protein-protein interactions by projection of interactions from three source organisms (yeast, human, thale cress) to *P. infestans*; co-expression, conservation of co-expression between *P. infestans* and *P. sojae* and phylogenetic co-occurrence in 51 species. Before the integration of the four data sources into a single network, these were scored based on their relative confidence using KEGG maps. **(B)** Number of orthologous groups between *P. infestans* and the three source organisms used for projecting physical interactions. **(C)** Projection of physical interactions via orthologous groups. In cases where the mapping was unclear, different genomic features such as co-expression and shared functional annotation were considered to disentangle these specific cases.

To integrate four complementary large-scale transcriptomic and comparative genomic data sets of gene-to-gene (protein-to-protein) associations we adopted a unified scoring schema (Figure 1A) that has been applied successfully in other eukaryotes [11,12]. This scoring schema is derived from Bayesian statistics and describes the log likelihood score (LLS) of association under given evidence and is corrected for the background expectation of association. Therefore, the LLS is proportional to the confidence of the given experiment to successfully recall known associations [11]; an LLS of 0 corresponds to random association. More importantly, this unified scoring schema allows accounting for the variability in the predictive quality between both binary data, such as predicted protein-protein interactions, as well as continuous data with an intrinsic scoring schema, such as the similarity between gene expression profiles. The continuous data is transformed into a range of LLS scores for different values of the intrinsic score (Material and Methods). Due to the lack of experimentally defined protein associations (positive set) and consequently also negative set in *P. infestans*, we approximated such sets using KEGG maps. Based on these approximations, we derived the prior odds (Additional file 1A), i.e. the ratio of probability of functional association and its negation without evidence, and the posterior odds, i.e. the ratio of probability of functional association and its negation given the evidence, for each dataset (Additional file 1B) and subsequently determined the LLS.

### Protein-protein interactions from three model organisms are projected to *P. infestans*

We projected a substantial number of physical interactions between protein pairs based on interolog mapping. To this end, we identified orthologs using an orthology detection algorithm (similar to Orthologous MAtrix OMA [21]) that we applied to an selection of 51 diverse eukaryotic species. We identified 3,507 orthologous groups (orthologous pairs + inparalogs) between *P. infestans* and at least one of the three genomes (*Homo sapiens* (human), *S. cerevisiae* and *A. thaliana*), of which 1,781 orthologous groups are shared between all four genomes (Figure 1B). Using the 3,507 orthologous groups, we projected protein-protein interactions from six different databases that aggregate information from *H. sapiens*, *S. cerevisiae* and *A. thaliana* to *P. infestans* (Additional file 1C). The information available from BioGRID and IntAct enabled discrimination between different levels of confidence*.* Since these interactions are mapped using orthology, some of the orthologous groups also include inparalogs and in some cases it is not directly obvious to which of the possible pairs the functional interaction would be most reliably mapped (see Figure 1C). These specific cases were disentangled using additional data considering overlapping and complementary functional characteristics, such as gene co-expression and cellular co-localization (Material and Methods).

**Figure 2:**
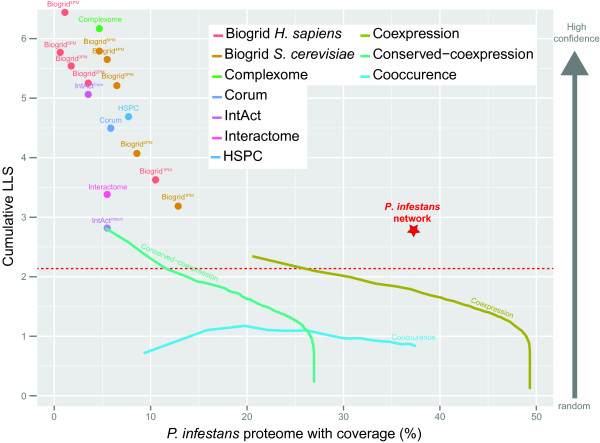
**Relationship between the log-likelihood score and the coverage of different data sources.** The relationship between log-likelihood score and coverage (percentage of *P. infestans* proteome) of the different data sources is displayed. Projected physical interactions are shown by dots and for BioGRID (number of supporting pubmed entries (PM), e.g. 1PM or 3PM) and IntAct (core or attachment) further subdivided based on the reliability. Continuous data sources, e.g. co-expression, are indicated as a line. The dashed red line shows the applied cutoff to include associations between proteins in the predicted *P. infestans* network. The coverage and log-likelihood score for the *P. infestans* association network is denoted with a red star.

All sixteen predicted protein-protein interaction networks, derived from the six different databases, have an LLS score that is higher than random linkage (LLS > 0), ranging from 2.8 (IntAct attachments) to 6.46 (BioGRID 4PM [human]), reflecting their high quality (Figure 2 and Additional file 1B). The ranking of the LLS for the KEGG and the alternative GO benchmark yields similar results (Material and Methods; Additional file 1B), even though the two benchmark sets are fairly independent: They share only 4,471 pairs of positives (12.6% of the positives annotated in KEGG) (Additional file 2), indicating the robustness of the LLS approximation of the mapped datasets.

### Complementary comparative genomic and gene expression data are integrated to predict functional associations in *P. infestans*

To also add complementary data to the mapped physical interactions from distantly related organisms, we used three other large-scale (comparative-) genomic data sets that could be indicative for the association between a pair of proteins (Figure 1A); (i) similarity in co-expression patterns, (ii) conservation of co-expression between co-expressed *P. infestans* genes and their orthologs in the soybean pathogen *Phytophthora sojae* and (iii) similarity in phylogenetic co-occurrence profiles measured in 51 eukaryotic species (Material and Methods).

These three genomic datasets score higher than random in our applied LLS scoring schema (Figure 2). As expected, their confidence is lower than the predicted protein interaction data, but the coverage of the proteome increases. Gene co-expression on its own has been shown to be a limited predictor of functional association; a correlation coefficient of 0.8 corresponds to an LLS of only ~0.71. However, if conservation of co-expression, orthologs of co-expressed genes are also co-expressed in a related species, is taken into account, this conserved co-expression is a high quality proxy for functional association [19]; a score of 0.8, which is approximated by the average of both correlation coefficients (Material and Methods) correspond to an LLS of ~1.25. This higher quality of conserved co-expression as a proxy for functional association, in return for a smaller coverage, is an observation that is also visible in our scoring schema (Figure 2).

### The prediction and initial survey of the functional association network of *P. infestans*

To obtain a comprehensive picture of functional associations, we integrated the four above described large-scale gene association data sets using a naïve Bayesian approach: we additively derived an LLS describing the combined evidence for association among pairs of proteins (Material and Methods). Each individual data source, even though less reliable by itself, adds evidence for the functional linkage of two proteins. Thereby, we unified these diverse lines of evidence into a comprehensive functional association network in *P. infestans* while simultaneously controlling quality (expressed by the associated LLS) and coverage of these predictions. We applied an LLS cutoff of 2.1 to each protein pair that corresponds to a conservative Pearson correlation coefficient for co-expressed gene pairs of ~0.94. This Pearson correlation coefficient was determined by the 99.9 quantile of the distribution of 100,000 random gene pairs. The LSS cutoff of 2.1 allows the inclusion of associations from genomic data sources if their score is above the LLS cutoff as well as the inclusion of lower scoring associations that require several independent lines of evidence to cumulatively pass the LLS cutoff.

**Figure 3:**
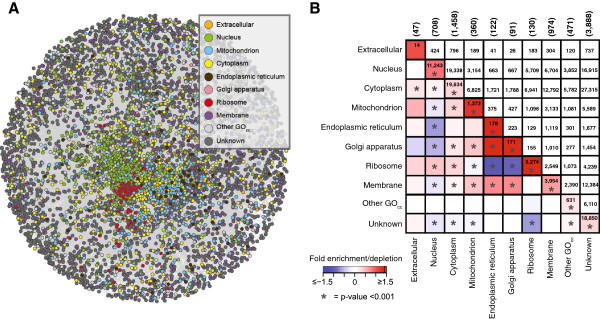
**The functional association network and the subcellular localization of its members. (A)** The predicted functional association network in *P. infestans*. Nodes, representing proteins, are colored according to their subcellular localization approximated by Gene Ontology term. **(B)** Correlation of subcellular localization with predicted protein associations. The log2-fold enrichment/depletion of protein pairs where both partners are predicted to reside in the same/different subcellular localization compared to the expected numbers is displayed by the heat map (lower half of the symmetrical matrix) (values saturate at ± 1.5); the corresponding raw numbers are shown in upper half. Significant enrichment/depletion (after multiple testing correction) is indicated by “*”. The total number of proteins predicted to reside in a particular subcellular localization is displayed in brackets above the plot.

The predicted network in *P. infestans* links 6,741 proteins (~37% of the predicted proteome), with 112,421 functional associations (Additional file 3). With a pairwise LLS cutoff of 2.1 for inclusion, the total confidence of the combined network is 2.75 (Figures 2; Figure 3A). As expected given the applied cutoff, 55% of the functional associations are in part derived from protein interaction data in other species; consequently the *P. infestans* functional association network is partially a physical interaction network. Moreover, 34,118 of these protein associations (~55%) have additional support based on other large-scale (comparative-) genomic data sets, giving further evidence for the robustness of the predictions. The network comprises 70 connected components (98% of proteins reside in the largest component; Figure 3A). A characteristic path length of 3.6, which is smaller than e.g. the overall protein-protein interaction network of *S. cerevisiae* but similar to the subset of essential proteins [22], and high clustering coefficient (0.27) are indicative of a dense network that reflects the homology-based projection of complexes and interactions.

Proteins that are part of the network show highly significant enrichment (all p-values <1e-7) in central cellular processes such as gene expression (GO:0010467), translation (GO:0006412), cellular localization (GO:0051641) and cell cycle (GO:0007049). The majority (51%) of proteins in the network is at least partially projected/included based on physical interaction which favors evolutionary conserved processes and hence explains the enrichment in core cellular processes. Nevertheless this information is useful as it provides further insights into the wiring of these core processes in *P. infestans*.

Given the nature of our analyses, the predicted network is mainly composed of evolutionary conserved processes (see above). Nevertheless, the network also includes few proteins with putative functions in pathogenicity or proteins that have been shown to induce defense responses in the host [6]; many of which are predicted to be secreted upon infection (Additional file 3). The network contains 364 secreted proteins; a 3.4-fold increase to the number we would have obtained if we only considered interactions derived by orthology projection of protein-protein interaction data. The RXLR- and Crinkler-effectors, two classes of host-targeted effectors that most likely promote infection of the host, are highly abundant in the proteome of *P. infestans* (596 RXLR- and 452 Crinkler-effectors) [1,23], and also occur 34 and 15 times, respectively, in the predicted network. The associations of these proteins with others are solely based on (conserved-) co-expression data, indicating involvement in the same process, without any evidence for potential physical associations. Two notable classes of highly abundant enzymes that are potentially linked to pathogenicity are glycoside hydrolases and peptidases [1,3,4,24]. We observed 53 glycoside hydrolases and 126 peptidases in the predicted network. This is a considerable increase of 2.6 and 1.8 fold, respectively, compared to a network that would only be based on projected physical data.

### The functional association network is enriched for co-localized protein pairs

Functionally associated proteins that show physical interaction are close together in the same subcellular compartment [25,26]. Subcellular localization therefore presents a suitable criterion to assess the biological significance of the predicted associations in *P. infestans* independently of the initial benchmark of (homology-based) KEGG pathways used to derive the LLS for each association*.* The network displays non-random distribution and local clustering of proteins with the same subcellular localizations, approximated by GO-cellular compartment (Figure 3A). To quantify this, we examined the enrichment/depletion of associations between proteins that are predicted to reside in the same/different subcellular compartment within the predicted functional association network (Figure 3B, Material & Methods). Associations between proteins with the same subcellular localization are significantly enriched, in agreement with observation on directly measured associations in other organisms and confirming the validity of our predicted network. Associations are enriched across compartments (meaning that partners are predicted to reside in different compartments) for the endoplasmic reticulum, the Golgi apparatus or membranes which is consistent with previous results for human proteins [26]. In accordance with the observations by Gandhi et al., proteins with predicted localization in the nucleus, the ribosome and to a smaller extent the mitochondrion do not tend to interact with proteins present in many other sub-compartments [26].

As homology is part of the initial source of the projected physical interactions – i.e. the LLS scoring via the KEGG benchmark as well as the prediction of subcellular localization via GO – we used two additional approaches to assess similarity in subcellular localization of predicted associations independent of homology. In the first approach, we divided the network into two components, one containing associations that are supported by at least one protein-protein interaction dataset (Additional file 4A), and the second, which is merely based on non-physical associations (co-expression, co-occurrence) (Additional file 4B). Both networks yield similar results in the (significant) enrichment of associated proteins predicted to co-localize. In a second independent approach, we used WoLF PSORT that predicts subcellular localization merely on sequence features and not homology [27]. Again, we found similar patterns of enrichments in associations between proteins with the same subcellular localization. Proteins residing in the nucleus and the mitochondrion showed depletions for associations with proteins predicted to reside elsewhere (Additional file 4C). These patterns are less pronounced, most likely because the prediction algorithm is not optimally trained for oomycete sorting signals. These independently derived similar patterns in enrichment and depletion support our predicted functional associations, even though experimentally verified associations, as present for other species, would provide a superior benchmark set to adjust confidence levels and assess the predicted associations.

### The compendium of protein complexes embedded within the functional association network

One of the major steps in understanding the function of a cell is to identify and determine the composition of its protein complexes. We mined the subset of the predicted functional association network that is supported by at least a single protein-protein interaction to derive protein complexes. We applied the ClusterONE algorithm that detects overlapping protein complexes in weighted networks by searching for sub-graphs that are characterized by many reliable interactions between proteins and separation from the remaining network [28]. In total, we detected 287 protein complexes covering 3,144 proteins (Additional file 5).

**Figure 4:**
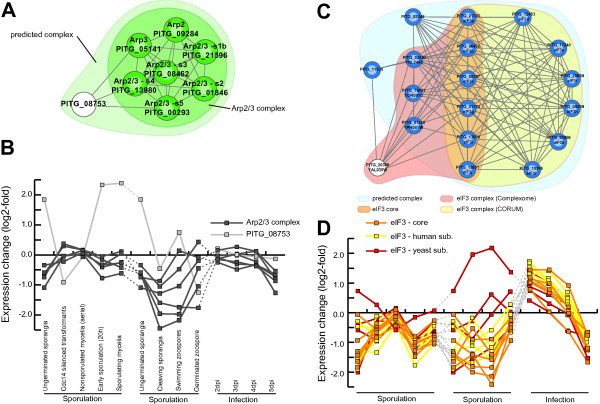
**Predicted Arp2/3 and eIF3 complexes in *P. infestans*. (A)** Automatically predicted Arp2/3 complexes (ClusterONE prediction in light green) include the seven conserved subunits of the eukaryotic Arp2/3 complex (Arp2, Arp3 and the five associated subunits; highlighted in dark green). **(B)** Gene expression of the predicted Arp2/3 complex (note: there is no gene expression data for Arp2/3 subunit 5 [BROAD:PITG_00293]). The log2-fold change in expression at different time points/developmental stages (averaged replicates) of three different gene expression experiments compared to the gene expression in mycelium/hyphae growth of the respective experiment is displayed in the graph. **(C)** Automatically predicted eIF3 complex (ClusterONE prediction in light blue), the annotated eIF3 complexes based on CORUM (yellow) and Complexome (red) database as well as the conserved eIF3 core (six subunits; orange). **(D)** Gene expression of the eIF3 core complex (orange) and the additional subunits predicted by either CORUM (yellow) or Complexome (red). The log2-fold change in expression at different time points (averaged replicates; description as in **(C)**) of three different gene expression experiments compared to the gene expression in mycelium/hyphae growth of the respective experiment is displayed in the graph.

Due to incomplete proteome annotation, members of a protein complex are unlikely to be identified by functional annotation (GO terms) alone. For example, the Arp2/3 complex, a central organizer of the actin filaments, contains seven subunits in yeast and human [29]. While its constitution can be completely retrieved in yeast and human based on its GO term (GO:0005885), the same is impossible in *P. infestans:* the annotation of the encoding genes is limited (only a single member of the Arp2/3 complex has this term) and higher-level terms such as cytoskeleton are too broad and retrieve too many results. The functional association network is therefore a necessary framework to predict and study the composition of protein complexes in *P. infestans.* Indeed, the Arp2/3 complex is one of the complexes we detected (complex 17). Besides Arp2 and Arp3, which have already been described [30], the detected complex contains the remaining five together with an additional subunit (Figure 4A). The genes encoding the seven subunits display a high degree of co-expression, whereas the additional protein, a tubulin-tyrosine ligase like protein (TTLL), is not co-expressed (Figure 4B), and therefore likely not part of the core Arp2/3 complex. In-depth investigation revealed that the associations to TTLL have been projected via a read-through transcript containing an Arp2/3 subunit and TTLL from human, underscoring the necessity to assess fusion transcripts in future analyses and to include gene expression data to validate and disentangle predicted protein complexes.

The analysis of another protein complex highlights the necessity of an integrative approach that combines different data sets from diverse organisms: The eukaryotic initiation factor 3 (eIF3) is among the largest translation initiation factors in eukaryotes [31]. Its conserved ‘core’ contains five essential (eIF3a, eIF3b, eIF3c, eIF3g and eIF3i) and one nonessential subunit (eIF3j) [31]. Only three of them could have been predicted in *P. infestans* based on GO terms. The eIF3 core is a subset of one of the detected complexes (complex 79; Figure 4C) that also contains several other subunits. The *H. sapiens* eIF3 complex described by the CORUM database contains six additional subunits, whereas the eIF3 complex described by the Complexome database contains four additional subunits, all of which have orthologs in *P. infestans*. Our predicted network unifies this information and consequently, the automatically inferred protein complex contains all these subunits, except a single protein from Complexome, and additional two, one of which are also eukaryotic translational initiation factors and hence likely functionally related. The genes encoding the eIF3 core proteins as well as the orthologs of the human complex are highly co-expressed and therefore likely forming a functional complex, whereas the orthologs of the yeast subunits, especially pronounced for eIF5 [BROAD:PITG_01255], show a lower level of co-expression (Figure 4D). The ATP-binding cassette protein RLI1 (yeast: [SGD:YDR091C]) is a conserved factor that has been implicated in several essential cellular processes such as translational initiation [32,33] and translational termination and recycling [34]. According to CORUM database, there is no interaction between human RLI (the ortholog to RLI1 in yeast) and eIF3 core factors, whereas the yeast complex in Complexome and consequently the predicted *P. infestans* network contain this experimentally determined interaction [32] (Figure 4C).

### Identification of functional modules during the development of *P. infestans*

Microarray technologies are a valuable source for the identification of genes involved in development and pathogenesis in *P. infestans*[1,15,35]. The interpretation of the results is challenging since a direct biological role for differentially expressed genes is not necessarily apparent, especially for uncharacterized gene products. The predicted functional association network provides a convenient framework to enhance the biological interpretation of gene expression data by placing functionally characterized and uncharacterized gene products in their cellular context.

**Figure 5:**
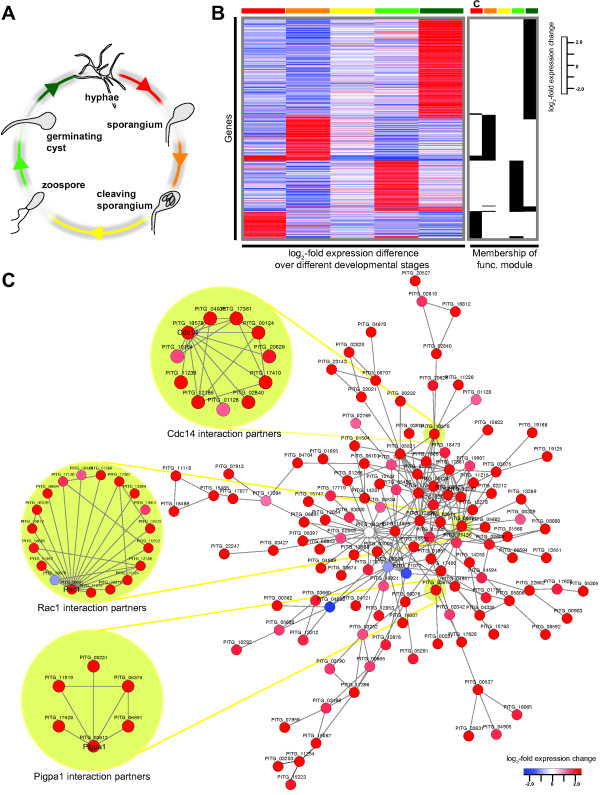
**Developmental stages of the asexual lifecycle of *P. infestans *and determined functional modules. (A)** Transition between five distinct developmental stages in the asexual lifecycle of *P. infestans*. Gene expression data of these stages are available [15] and were used to assess gene expression changes between the transitions. Transitions between different developmental stages are colored coded. **(B)** The gene expression changes and the membership of all 825 genes predicted in the five functional modules are displayed. The heat map shows the gene expression changes (log2) for the transitions of two subsequent life stages (same color code as in A; heat map saturated at ± 2.5). Presence (black) or absence (white) of genes in a functional module is highlighted next to the heat map and membership is indicated by color code. C refers to the sporulation module as described in **(C)**. **(C)** Determined functional module of genes up-regulated during sporulation and their predicted associations. Examples discussed within the text and their directly associated proteins are highlighted with light green. The nodes are colored according to the fold change in expression in sporangia compared to hyphae (same scale as in B).

We aimed to apply the predicted network to identify functionally related subsets of differentially expressed genes at defined time points in the lifecycle of *P. infestans* (Figure 5A). To prevent circularity in the analysis, we excluded associations within the network that were only supported by gene expression data, leaving us with a network of 62,000 associations between 3,500 proteins. We used the algorithm HEINZ [36] that automatically finds the subset of up-regulated genes that are also interconnected by a significant amount of associations. Such an ensemble of genes is referred to as a functional module, and allows identifying and studying proteins likely involved in a defined process and their associations. We studied five different time points during the asexual development of *P. infestans*[15]: *in vitro* growing nonsporulating hyphae, sporangia, cleaving sporangia, swimming zoospores and germinating zoospore cysts that contain specialized infection structures called appressoria (Figure 5A). We defined differentially expressed genes at each transition and subsequently detected functional modules (Figure 5A and B).

Each developmental transition is represented by a functional module of associated differentially expressed genes. The modules display little overlap and a distinct pattern of gene expression changes (Figure 5B). They vary in size, ranging from nearly 400 members at the transition from germinated cyst to hyphal growth, to only two members at the transition from cleaving sporangia to swimming zoospores (Additional file 6). The latter transition contains only very few up-regulated genes (FDR 0.05) in the predicted network which is the reason for the small size of the module. Interestingly, the functional module at the former transition is enriched for proteins with a predicted function in proteolysis (GO:0006508; p-value << 1e-4), including twelve, mostly intracellular, peptidases. In contrast, the transition from hyphae to sporangia is significantly (p-value < 1e-3) enriched for regulation of biological process (GO:0050789) and in particular signal transduction (GO:0007165). Among other proteins involved in regulation, we also found ten kinases. Four of these are also found in the functional module of the subsequent transition from sporangium to cleaved sporangium, a module that contains 17 kinases. Kinases have been reported to be among the genes with the highest fold expression change in this transition [15]. Oomycetes contain an extensive repertoire of these central regulators [24,37]. The high abundance of kinases in functional modules points to their prominent role in regulation of sporangium formation. The associations of and amongst these kinases present important novel information that would not have been available using gene expression data alone.

### The sporangia formation module contains genes encoding known and novel proteins, and novel associations

To highlight the merit of the functional association network as a framework to study gene expression and the predicted associations between co-regulated proteins, we further studied the initial phase of sporulation. In *Phytophthora* this major transition leads to the formation of sporangia, asexual spores that can either germinate directly and infect the host, or develop into a zoosporangium which cleaves and releases multiple zoospores that function as infectious propagules (Figure 5A). We identified a module that contains 128 interconnected proteins of which 124 are encoded by up-regulated genes during sporangium formation (Figure 5C). This functional module is significantly enriched (p-value <0.05) for proteins with predicted functions in signal transduction (GO:0007165), cell differentiation (GO:0030154) and developmental processes (GO:0032502). Interestingly, our predicted module contains most proteins known to be involved in sporangia formation, but also many novel interactors that have not yet been associated with this important process.

In the predicted functional module we observed Pigpa1 [BROAD:PITG_03612] and Pigpb1 [BROAD:PITG_06376], the alpha and beta subunit of the heterotrimeric G-protein. Both encoding genes are up-regulated early during spore formation [38]. Whereas *Pigpb1* silenced mutants have malformed sporangia and very few asexual spores [39], *Pigpa1* silenced mutants show altered zoospore mobility, reduction in zoospore release and appressorium formation [40]. One of the predicted interaction partners of both Pigpa1 and Pigpb1 is a Rac1 homolog [BROAD:PITG_06691]*,* a small GTPase of the Ras-like superfamily. Its role as a central regulator is corroborated by several predicted interaction partners: eukaryotic protein kinases such as mitogen-activated kinases [BROAD:PITG_02212/BROAD:PITG_17361/BROAD:PITG_12186] or the phosphatidylinositol-4-phosphate-5-kinase [BROAD:PITG_15552]. Next to Rac1, we observed other signal transduction components related to the Ras superfamily of GTPases such as ARF-like [BROAD:PITG_13269] and Rab [BROAD:PITG_19907/BROAD:PITG_17136], highlighting the importance of these signaling proteins and the associations of these novel candidates for spore formation.

In the functional module we also observed the phosphatase Cdc14 [BROAD: PITG_18578]. In eukaryotes, it plays a role in a variety of processes including cell cycle regulation and termination of mitosis. In contrast to its orthologs, *Cdc14* in *P. infestans* is specifically expressed during sporulation, and has a central role in spore formation [41]. It also does not seem to be involved in the regulation of mitosis during normal growth, even though it complements the function of Cdc14 in yeast [41] and therefore might still maintain this regulatory role during sporulation [42]. Additionally, recent evidence points to a possible role of *P. infestans* Cdc14 in the development of the flagellum due to its co-localization with the known basal body marker DIP13 (deflagellation-inducible protein; [BROAD:PITG_13461]) [42]. Even though *Cdc14* and *DIP13* show considerable (conserved) co-expression (Pearson correlation coefficient 0.76), this evidence is insufficient to infer association within the framework of our network. Interestingly, Cdc14 is predicted to interact with a 4.3-fold (log2) up-regulated tyrosine kinase [BROAD:PITG_17410] (Figure 5C). We observed an association between this kinase and DIP13 (not based on physical evidence; Additional file 3), therefore indirectly linking DIP13 to Cdc14 as initially suggested by the co-localization studies by Ah-Fong and colleagues [42].

The up-regulated Cdc14 interaction partners within the functional module include several other kinases such as the 2.5-fold (log2) up-regulated Ser/Thr kinase [BROAD:PITG_00124]. Interestingly, we predicted a novel association between Cdc14 and NIFC1 [BROAD:PITG_11238], a protein that contains a nuclear LIM interactor-interacting factors domain and is reported to be involved in transcriptional regulation [43]. *NIFC1* is highly expressed during zoospore-formation (cleavage) [15,43], whereas *Cdc14* is expressed early during sporangium formation and maintains a high expression level during zoospore-formation. Together with the predicted association between Cdc14 and the sir2-like histone deacetylase [BROAD:PITG_10164], these interactions imply a role of Cdc14 as a transcription regulator to reprogram gene expression during zoospore formation.

We highlighted how the predicted functional association network serves as a valuable framework for the analysis of gene expression data. The delineation of functional modules generates a concise set of candidates and their associations for further studies. The sporangia formation module illustrates this nicely: firstly we identified proteins that have been already experimentally linked to this transition, e.g. Cdc14 and Pigpb1. Subsequently, we were able to place these in their wider cellular context allowing the identification of directly associated proteins. Since many of these have only putative functions (~50%) or are without functional annotation (~28%) the functional network approach used in this study revealed interesting novel candidates that may play central roles in sporangia formation.

## Conclusions

Proteins rarely act alone. They interact either directly or indirectly with other proteins to synergistically mediate biological functions. So far, hardly anything is known about this complex interplay between proteins in oomycetes. The only large-scale experimental study in oomycetes investigated the interactions between effector proteins produced by the downy mildew *Hyaloperonospora arabidopsidis* with known proteins from the *A. thaliana* (thale cress) immune system [44]. Although this study emphasized the importance of functional association data, it solely addressed associations within the plant cells and not in the pathogen.

As an initial step on the way to fully expose the ensemble of all functional associations between proteins, we here present the first functional association network *in P. infestans*. We combined available genomic, transcriptomic and comparative genomic data to predict associations (interactions) between protein pairs resulting in a comprehensive network of gene associations that covers 37 percent of the predicted proteome. As expected, this number is lower than previous studies in *S. cerevisiae*[11] or *A. thaliana*[12], reflecting the relative paucity of data, especially for large-scale descriptive data such as gene expression, in *P. infestans* compared to these well studied model organisms. The majority (>50%) of the associations are predicted using conservation (interolog mapping) and therefore our network is biased for core cellular processes. Consequently, we observed only very few genes with a proposed role in pathogenicity (RXLR, Crinkler or hydrolases) and those we do observe are mainly associated by integration of complementary comparative genomics and expression data. Nevertheless, the availability of associations for a considerable fraction of the predicted proteome is crucial to provide insights into functional genomics in this group of organisms.

We balanced the coverage with an acceptable level of confidence given all available large-scale data and our *in silico* benchmark. The lack of experimentally confirmed benchmark sets in *P. infestans* limits a completely independent assessment of our prediction. In the future, more complementary gene expression data will most likely be available and consequently, together with experimentally determined interactions in *P. infestans* and closely related species, the genome-wide prediction of functional associations will be enhanced. This is of special importance for pathogenicity related genes that are currently underrepresented in our predicted network due to the limitations (quantity, coverage and divergence) of the currently available gene expression data.

We showed that proteins that are predicted to be functionally associated are enriched to reside in the same, or related, cellular sub-compartments, further validating the biological coherence of our predictions and the merit of the applied integrative approach. We exemplified the usability of the predicted functional association network on two examples: We automatically determined protein complexes and subsequently studied their constitution; an analysis that is not possible by just applying functional annotation to the genome. Moreover, we highlighted how the availability of the functional association network together with gene expression data allowed us to predict modules of functionally related genes during distinct phases of development. We exemplified this by analyzing the sporulation module that contained several experimentally characterized proteins such as Cdc14 and Pigpb1. The predicted physical interaction partners to these well-described proteins allowed us to place a concise set of candidates into a prominent role in sporangia formation.

Our study created a so far lacking addition to the growing genomic resources in the plant pathogenic model organism *P. infestans*. We demonstrated that these data are needed to further improve the ability to retrieve biological knowledge from large-scale data such as microarrays, RNA-seq or (phospho-) proteomics. The availability of the predicted functional association network allows a gradual transition from a single gene perspective to a more comprehensive understanding of the complex biology of *P. infestans* and other oomycetes.

## Material and methods

### Prediction of orthologs between 51 eukaryotic species

We defined the groups of orthologs for a set of 51 eukaryotic species that were selected based on the taxonomic diversity. The orthologous groups were computed following an OMA (Orthologous MAtrix)-like algorithm [21,45] which was adjusted to the specific requirements of the analysis. To also identify weaker similarity between sequences we modified the following steps: (i) the minimal alignment score for potential orthologs was reduced to 130, (ii) the minimal alignment coverage was reduced to 40% in the first clustering step (assembling doubly-connected components, as opposed to cliques in the original OMA algorithm) and (iii) alignments with only 25% sequence coverage were added to the best matching cluster. We empirically determined the necessary cutoff values to maximize the inclusion of distant homologs while at the same time avoiding the excessive clustering of paralogs. This approach clustered in total 644,999 proteins into 58,533 orthologous groups. Each group is intended to represent all extant descendants from a single gene in the last common ancestor of eukaryotes; or, for a gene invented later, all descendants of that gene.

### Interolog transfer of protein-protein interactions

We retrieved in total sixteen protein-protein interaction networks from six different sources (Additional file 1C). Three of these data sets were subsequently subdivided, either to account for different levels of confidence expressed by the number of distinct publications (1PM-5PM) confirming an interaction (BioGRID) or to distinguish between core and attachments (IntAct). BioGRID interactions were mainly based on protein-protein interaction, however if at least a single publication reported the physical associations, also genetic interactions were considered to enhance the support for the specific association (2PM-5PM).

Interactions from the source databases were first mapped to the human Ensembl, yeast and *Arabidopsis* identifiers and subsequently projected from the source species to *P. infestans* using the identified orthologous groups. Since orthologous groups can also contain inparalogs, both in the source (*H. sapiens*, *A. thaliana* and *S. cerevisiae*) and in *P. infestans*, we excluded all genes from the mapping with an alignment score to the source gene of less than 75% of the best matching inparalog, assuming that larger differences might be indicative of neo-functionalization of the paralog. If the mapped pairs still included inparalogs in *P. infestans*, we disentangled these specific cases by applying four different functional criteria to define which of the *P. infestans* proteins most likely retained the interaction. An interaction between two proteins is retained if both proteins (i) are on the same Kyoto Encyclopedia of Genes and Genomes (KEGG) map [46], (ii) have protein domains that are known to mediate protein-protein interactions, (iii) share a common Gene Ontology [47] (GO) term (biological process or cellular component) at a depth of level ≤ 6 or ≤ 5, respectively, (iv) share a common GO term (biological process or cellular component) at a depth of level ≤ 4 and their expression profiles have a Pearson correlation coefficient ≥ 0.4. If none of these criteria was applicable we chose the protein with the highest similarity to the source protein so that we kept at minimum a single interaction between a set of orthologous groups.

The details of these four criteria to disentangle inparalogs in *P. infestans* are as follows: (i) To define pairs that are on the same KEGG map, we retrieved 94 predicted KEGG maps for *P. infestans* from the KEGG database (01.05.2012; excluding maps pif01100 and pif01110) that contained in total 1,329 proteins from *P. infestans* (7.5% of the predicted proteome). (ii) Protein domains that are predicted to mediate protein-protein interactions are retrieved from 3did (03.05.2012). Protein domains are predicted for the proteome of *P. infestans* using hmmer3 [48] (gathering cutoff) and a local Pfam-A database (v26) [49]. (iii) We predicted the GO terms for all predicted proteins in *P. infestans* using the BLAST2GO algorithm (default parameters) [50]. Since GO is an acyclic graph, we first searched within each of the two domains (biological process or cellular component) for common GO terms between the two potentially interacting proteins. For all possible combinations of GO terms between the two proteins, we first searched all possible paths for common GO terms that minimize the distance to the initial GO term. If more than a single GO term is equally distant to the initial GO term, we chose the common term that minimized the distance to the root of the ontology. Subsequently, the depth of the common GO term that is shared between the proteins, which can be seen as a measure of functional similarity, is assigned to the pair by calculating the shortest path to the root of the ontology (Additional file 2). (iv) In addition to the approach outlined in (iii), we added gene expression data as a complementary feature (details to the gene expression analysis can be found below). We calculated Pearson correlation coefficients of the expression profiles between two pairs and kept an interacting pair if both the depth cutoff and the correlation cutoff were reached. Suitable cutoffs for the GO depth and the Pearson correlation in (iii) and (iv) were determined by maximizing the positive predicted value and the accuracy while minimizing the false discovery rate for 1,000 randomly picked positive pairs as defined by KEGG (see above) and 1,000 random gene pairs or 500 pairs for (iii) and (iv), respectively.

### Functional interactions by additional comparative genomics data

To define the functional interaction network in *P. infestans*, we added complementary data next to the predicted protein-protein interactions. We used (i) co-expression, (ii) conserved co-expression and (iii) co-occurrence to define these additional functional associations between two genes. (i) Publicly available gene expression data for *P. infestans* was extracted from NCBI Gene expression omnibus [51] with the accessions GSE9623 (Affymetrix), GSE13580 (Affymetrix), and GSE14480 (NimbleGen). The Affymetrix data were normalized using MAS5 and the log2 of the expression intensities was computed using Bioconductor (Affy package) [52]. Replicates were averaged and the resulting gene expression vector was normalized calculating the Z-score per unigene. Because the Affymetrix chip was designed prior to the availability of the genome sequence of *P. infestans*, we mapped the unigenes that have been used in the chip design to the transcripts derived from the *P. infestans* genome. We only considered the best hits of each unigene to the transcript set (blastn [53], evalue cutoff 1e-20, ≥ 95 percent identity). If several independent unigenes have the same transcript as their best hit we assigned the most C-terminal unigene to this transcript, since these unigenes tend to have the highest expression values. Normalized target intensities (log2) were extracted from the NimbleGene data, replicates were averaged and Z-scores were calculated. The three independent experiments (the union of the genes in the three experiments is 8,749 genes) were combined to compute pairwise Pearson correlation coefficients between all genes. (ii) To predict pairs of proteins that are encoded by conserved co-expressed gene pairs in *P. infestans*, we used defined orthologs between *P. infestans* and *P. sojae* as outlined above using a confined species selection. Furthermore, we used three publicly available gene expression data sets for *P. sojae* GSE15100 (Affymetrix), GSE22978 (Affymetrix) and GSE735084 (RNA-seq). The analysis of the two Affymetrix expression sets was conducted as described above, however, before normalization all non-*P. sojae* probes (the vast majority for this array) were removed. The RNA-seq derived gene expression intensities were log2 transformed and otherwise treated similarly to the microarray experiments (see above). Pearson correlation coefficients of the normalized (Z-score) and subsequently combined gene expression values were calculated for all genes (union of the thee experiments, i.e. 7,716 genes). A single unified score for each conserved co-expressed gene pair was derived by rescaling (between 0 and 1) the averaged Pearson correlation coefficients of the gene pair in *P. infestans* and the orthologous gene pair in *P. sojae.* The average Pearson correlation was calculated after applying a Fisher’s Z-transformation to the individual correlation coefficients. (iii) We predicted putative pairs of functionally associated proteins by comparing the phylogenetic profiles of all genes with at least one gene loss during their evolutionary history. The similarity between profiles was measured by reconstructing the gene gain and loss events within an orthologous group over all 51 eukaryotic species. We ignored duplications, since the presence/absence of a gene within a genome was taken into account. We used ‘partial correlations’, as described by Cordero et al. in detail [54], to compare the gains and losses assigned to the branches of the species tree. The ‘partial correlation’ is based on the Pearson correlation coefficient of the events, but corrected against genome-wide trends such as whole-genome duplications or genome streamlining. We based the threshold on the 99 percent quantile of the partial correlation, estimated from 200,000 random pairs.

### Bayesian integration of distinct data sources

We integrated the different data sources by applying a scoring system that is derived from Bayesian statistics followed by a Bayesian integration approach as outlined by Lee et al. [11]. Briefly, we calculated for each data source the log likelihood score (LLS) (log odd ratio) that two proteins are functionally linked, defined as LSS = log_e_(O_Posterior_ / O_Prior_). The LLS was calculated based on the prior odds (O_Prior_), describing the ratio of probability of functional linkage and its negation without evidence, and the posterior odds (O_Posterior_), describing the ratio of probability of functional linkage and its negation under the given evidence. The prior odds can be estimated by the number of protein pairs that share a defined functional annotation, e.g. being on the same KEGG map, and the number of protein pairs that do not share the functional annotation, e.g. residing on two different KEGG maps. Similarly, we derived the posterior odds by estimating the number of protein pairs that share or do not share functional annotation and are supported by the given evidence. We mainly used KEGG to estimate the prior odds and the posterior odds for each dataset. Alternatively, we also used GO – 6th level (biological process) to derive prior odds and compared these to the KEGG based results. If the dataset is discrete (e.g. protein-protein interactions) a single LLS is determined (Additional file 1b). If the dataset has a continuous scoring schema, e.g. Pearson correlation coefficient for the co-expression data, we first determined a mapping function to re-score the initial score to the corresponding LLS similar to Lee et al. [11]. Therefore we ranked the original initial scores (e.g. Pearson correlation coefficients) and calculate the LLS for protein pairs in equally sized bins (bin size 20,000 (conserved co-expression/co-occurrence) and 80,000 (pairs co-expression)). Subsequently, we performed a non-linear regression on the initial score and the calculated LLS to determine the coefficients of the fitted function (Additional file 7). This function is used to subsequently re-score the initial score of a pair of proteins to our LLS schema, thereby converting independent scoring schema into a single unified LLS schema. The combined LLS of all available evidences for an association between a pair of genes/proteins was calculated using a naïve Bayesian approach: LLS_sum_ = SUM(LSS)_PPI (excl. Biogrid)_ + max(LSS)_BioGrid Human_ + max(LSS)_BioGrid Yeast_ + LLS_CE_ + LLS_CC_ + LLS_CO_. If the summed LLS of a pair of proteins was smaller than the cutoff, the association was not reported. We also implemented and evaluated a weighted Bayesian approach as outlined by Lee et al. (2004) [11]. For our data, this integration approach results in a marginal increase in confidence combined with substantial reduction in coverage.

### Enrichment/depletion of subcellular localization of protein pairs

We assessed the enrichment/depletion of the shared subcellular localization between pairs of associated proteins as outlined by Gandhi et al. [26]. Briefly, we calculated the fold enrichment/depletion of the number of observed edges between proteins of a certain subcellular localization (GO cellular compartment; a single protein can be annotated to be localized in more than one cellular compartment), e.g. number of edges between proteins where one partner is annotated as residing in the nucleus and the other in the endoplasmic reticulum, compared to the expected number of edges based on random networks that maintained the protein annotation, the degree for each protein (number of associations) and the total number of edges (see Gandhi et al. for details [26]). The statistical significance was assessed using a Poisson distribution and the resulting p-value was corrected for multiple testing.

We independently predicted subcellular localization using the WoLF PSORT algorithm that uses sequence features and not homology to assign localization [27]. We used both animal and fungi presets, assigning subcellular localization to protein upon agreement, otherwise to ‘unknown’. Enrichment and depletion was otherwise calculated as described above.

### Functional modules

Functional modules, i.e. maximally co-regulated sub-networks under a defined condition, were predicted based on differentially expressed genes between two conditions assessed by limma [55]. The functional module was identified in a subset of the functional network, excluding associations that were merely supported by gene expression. Moreover, only the proteins whose genes have corresponding expression values and were part of the largest component within the sub-network were considered. The functional module within each sub-network was identified using BioNet [36], where the p-values obtained from limma were scored using a fitted beta-uniform mixture model and a false discovery rate of 0.01. We were only interested in up-regulated modules during the defined condition, consequently we set all scores of proteins to –abs(S) when the gene expression difference expressed as fold (log2) was smaller than 0, thereby only allowing inclusion of these nodes in the functional module if they connect high scoring nodes.

## Competing interest

The authors declare that they do not have any competing interest.

## Authors’ contribution

MFS, FG and BS conceived the study. MFS and AS analyzed the data. MFS, FG and BS drafted the manuscript. All authors read and approved the final manuscript.

## Supplementary Material

Additional file 1 **Calculation of prior/posterior odds and used protein-protein interaction data.** The table contains (a) the calculated prior odds of association based on the KEGG and GO benchmark, (b) the calculated posterior odds and log-likelihood scores for the protein-protein interaction data sets and (c) the used data sources to project protein-protein interactions form different source organisms to *P. infestans*. Click here for file

Additional file 2 **Determination of positive and negative sets for benchmark.** Approximation of positive and negative associations using (a) KEGG and GO (biological process). (b) The number of proteins that are annotated by KEGG and GO. (c) The number of determined protein pairs and the fraction of positives. Click here for file

Additional file 3 **Log-likelihood evidence for the association between protein pairs.** All log-likelihood evidence for the association between protein pairs in the functional association network. The contribution of each individual evidence to the combined LLS score is indicated. Moreover, functional annotations (secretome, RXLR, Crinkler, glycoside hydrolase, peptidase, Gene Ontology (biological process)) are displayed for each protein in the predicted association network. Click here for file

Additional file 4 **Correlation of sub-cellular localization with predicted protein associations.** The figures displays the log_2_-fold enrichment/depletion of protein pairs where both partners are predicted to reside in the same/different sub-cellular localization compared to the expected numbers. We discriminated between associations that have predicted protein-protein interactions as a source of evidence (a) and associations that were merely predicted by co-expression, conserved co-expression and co-occurrence (b). Panel (c) shows the same information, however the sub-cellular localization was predicted using WoLF PSORT (Material and Methods). Enrichment/depletion is displayed by the heatmap (lower half of the symmetrical matrix) (values saturate at +− 1.5 or +−1 for WoLF PSORT); the corresponding raw numbers are shown in upper half. Significant enrichment/depletion (after multiple testing correction) is indicated by asterisk. The total number of proteins predicted to reside in a particular sub-cellular localization is displayed in brackets above the plot. Click here for file

Additional file 5 **Inferred protein complexes in *P. infestans*.** This table lists the automatically inferred protein complexes in *P. infestans* using ClusterONE alongside GO annotation of the proteins. Click here for file

Additional file 6 **Functional modules during the asexual development of *P. infestans.*** This table lists the gene ids and associations between members of functional modules for different transitions during the asexual development of *P. infestans*. Upregulated genes that are part of the functional module are indicated in red, downregulated in green. Click here for file

Additional file 7 **Mapping of continuous scores to the unified log-likelihood schema.** The figure displays the mapping of intrinsic continuous scores from (a) co-expression, (b) conserved co-expression and (c) phylogenetic co-occurrence to the unified log-likelihood schema. The derived mapping function (based on non-linear regression; Material and Methods) for each mapping is shown. Click here for file
