# Supplementary material for: A predicted functional gene network for the plant pathogen Phytophthora infestans as a framework for genomic biology
Source: BMC Genomics. 2013 Jul 17;14:483. doi: 10.1186/1471-2164-14-483 (PMC3734169; doi:10.1186/1471-2164-14-483)
Supplement: Additional file 2 — Determination of positive and negative sets for benchmark. Approximation of positive and negative associations using (a) KEGG and GO (biological process). (b) The number of proteins that are annotated by KEGG and GO. (c) The number of determined protein pairs and the fraction of positives. [file 1471-2164-14-483-S2.pdf]

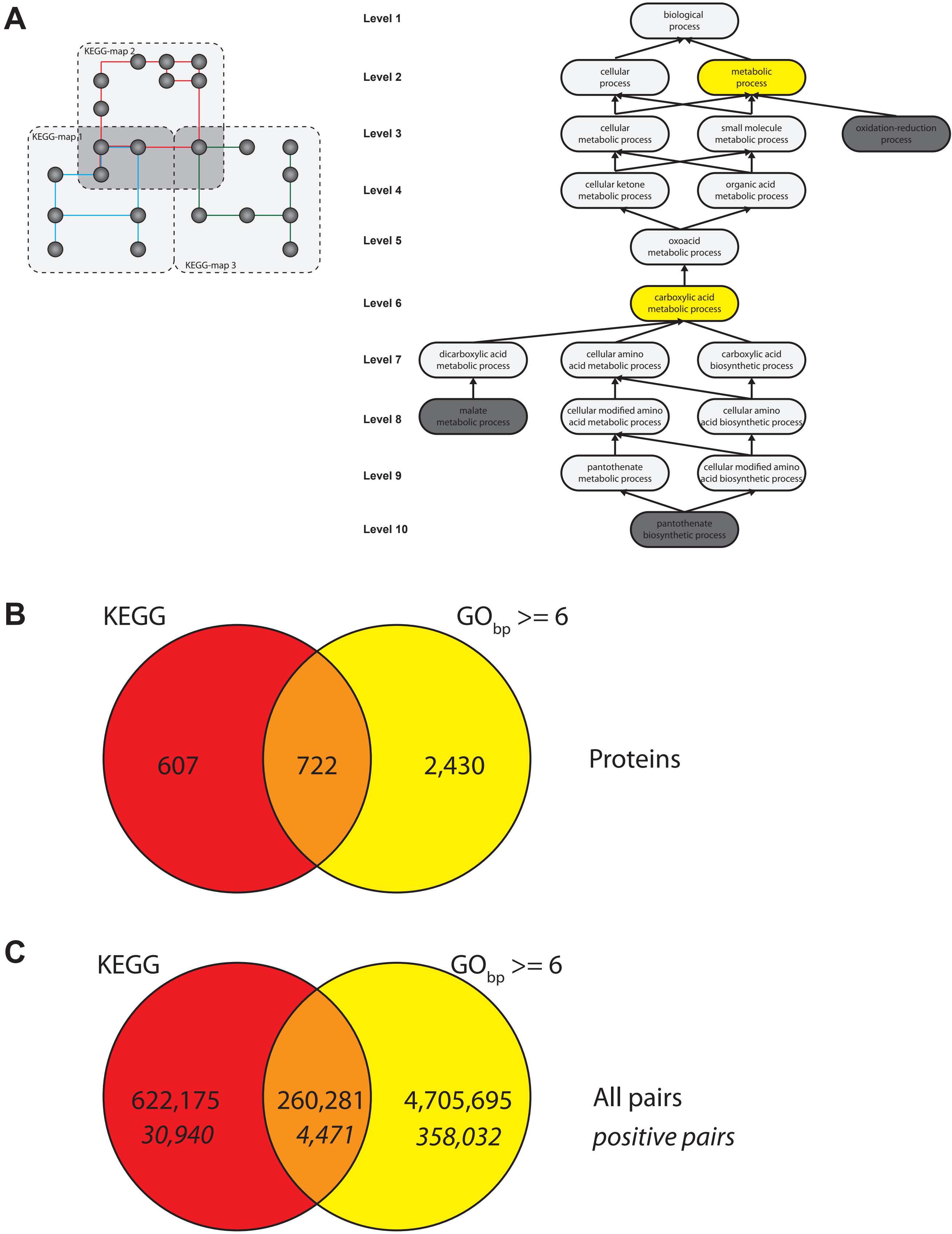

A
KEGG-map 2
KEGG-map 1
KEGG-map 3
Level 1
Level 2
Level 3
Level 4
Level 5
Level 6
Level 7
Level 8
Level 9
Level 10
biological process
cellular process
metabolic process
cellular metabolic process
small molecule metabolic process
oxidation-reduction process
cellular ketone metabolic process
organic acid metabolic process
oxoacid metabolic process
carboxylic acid metabolic process
dicarboxylic acid metabolic process
cellular amino acid metabolic process
carboxylic acid biosynthetic process
malate metabolic process
cellular modified amino acid metabolic process
cellular amino acid biosynthetic process
pantothenate metabolic process
cellular modified amino acid biosynthetic process
pantothenate biosynthetic process
B
KEGG
GObp >= 6
607
722
2,430
Proteins
C
KEGG
GObp >= 6
622,175
30,940
260,281
4,471
4,705,695
358,032
All pairs
positive pairs
