# Supplementary figures and images for: A predicted functional gene network for the plant pathogen Phytophthora infestans as a framework for genomic biology
Source: BMC Genomics. 2013 Jul 17;14:483. doi: 10.1186/1471-2164-14-483 (PMC3734169; doi:10.1186/1471-2164-14-483)

A

only PPI

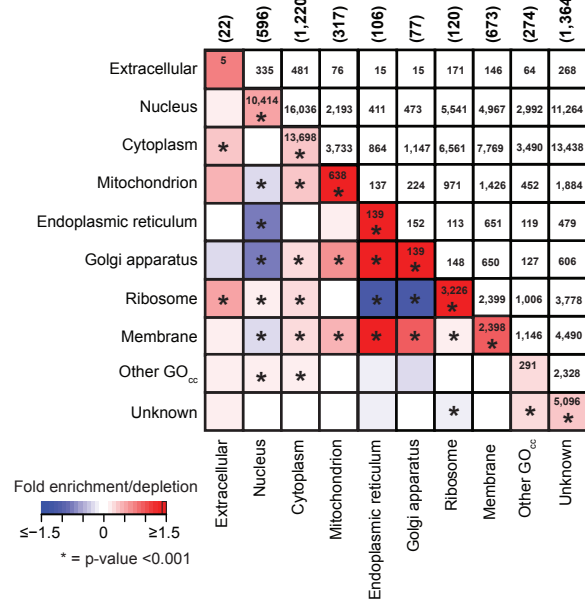

B

without PPI

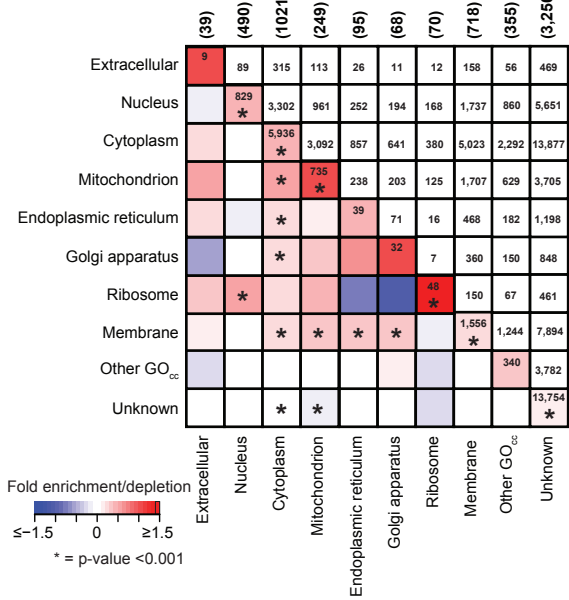

C

full network

only PPI

without PPI

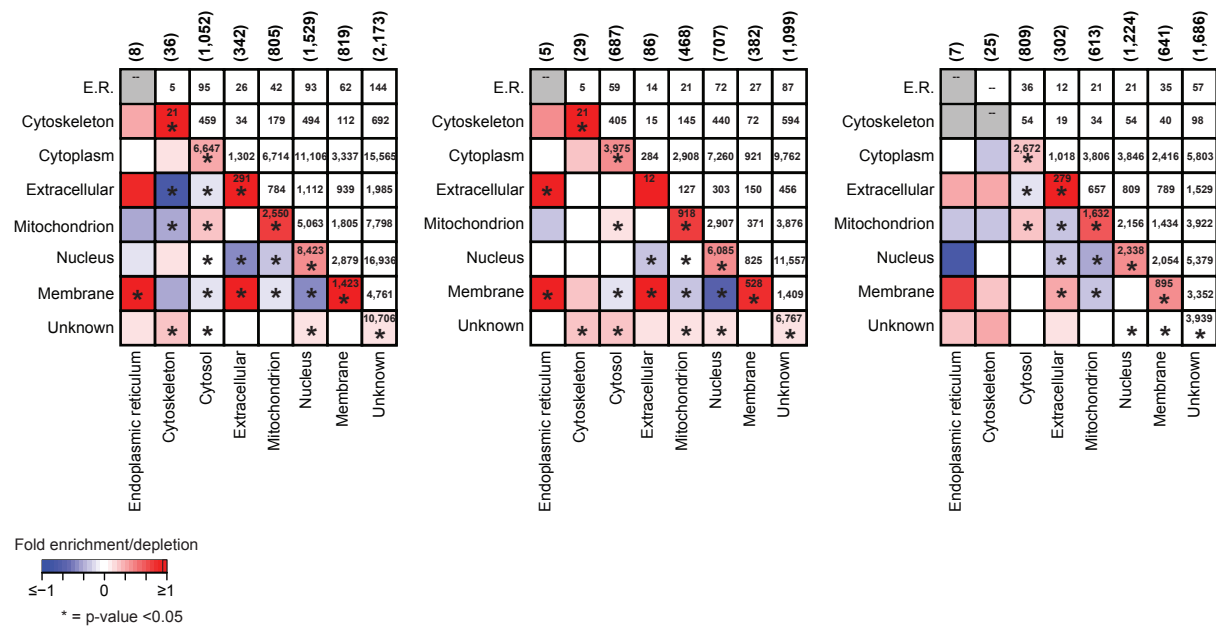

Supplement: Additional file 4 — Correlation of sub-cellular localization with predicted protein associations. The figures displays the log2-fold enrichment/depletion of protein pairs where both partners are predicted to reside in the same/different sub-cellular localization compared to the expected numbers. We discriminated between associations that have predicted protein-protein interactions as a source of evidence (a) and associations that were merely predicted by co-expression, conserved co-expression and co-occurrence (b). Panel (c) shows the same information, however the sub-cellular localization was predicted using WoLF PSORT (Material and Methods). Enrichment/depletion is displayed by the heatmap (lower half of the symmetrical matrix) (values saturate at +− 1.5 or +−1 for WoLF PSORT); the corresponding raw numbers are shown in upper half. Significant enrichment/depletion (after multiple testing correction) is indicated by asterisk. The total number of proteins predicted to reside in a particular sub-cellular localization is displayed in brackets above the plot. [file 1471-2164-14-483-S4.pdf]

**A** best fit:  $0.001309 * \exp(7.87693 * x)$

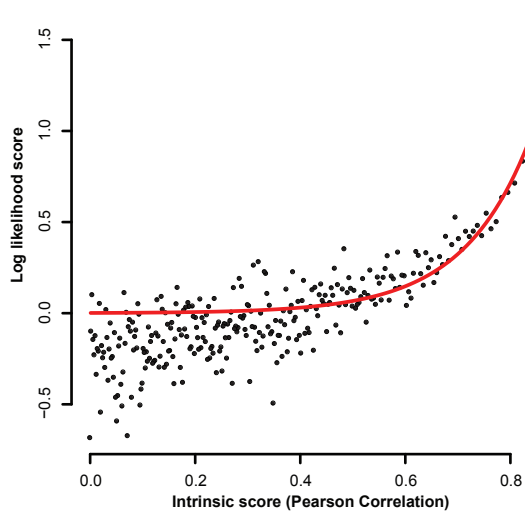

**B** best fit:  $0.006419 * \exp(6.5814 * x)$

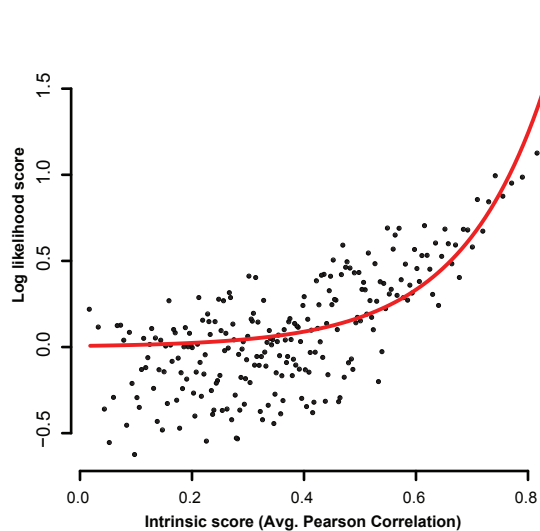

**C** best fit:  $1.21 / (1 + \exp(6.76 - (10.94 * x)))$

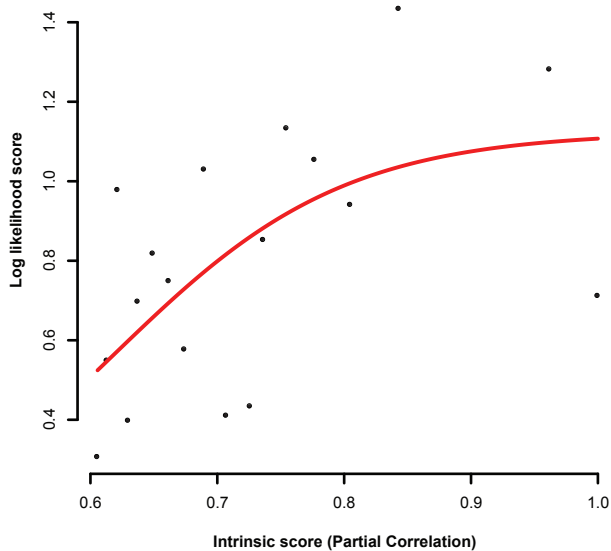

Supplement: Additional file 7 — Mapping of continuous scores to the unified log-likelihood schema. The figure displays the mapping of intrinsic continuous scores from (a) co-expression, (b) conserved co-expression and (c) phylogenetic co-occurrence to the unified log-likelihood schema. The derived mapping function (based on non-linear regression; Material and Methods) for each mapping is shown. [file 1471-2164-14-483-S7.pdf]
